# Arm Circumference-to-Height Ratio as a Situational Alternative to BMI Percentile in Assessing Obesity and Cardiometabolic Risk in Adolescents

**DOI:** 10.1155/2018/7456461

**Published:** 2018-09-06

**Authors:** Wasantha Jayawardene, Stephanie Dickinson, David Lohrmann, Jon Agley

**Affiliations:** ^1^Applied Health Science, School of Public Health Bloomington, Indiana University, 116 SPH-B, 1025 E. 7th Street, Bloomington, IN 47405, USA; ^2^Institute for Research on Addictive Behavior, School of Public Health Bloomington, Indiana University, 501 N. Morton St., Suite 110, Bloomington, IN 47404, USA; ^3^Epidemiology and Biostatistics, School of Public Health Bloomington, Indiana University, C-003 SPH-B, 1025 E. 7th Street, Bloomington, IN 47405, USA

## Abstract

**Objective:**

To determine whether arm circumference-to-height ratio (AHtR) predicts adolescents' cardiometabolic risk and how its predictive statistics compare to those of body mass index (BMI) percentile.

**Methods:**

Pooled data for adolescents (*N* = 12,269, 12–18 years) from the National Health and Nutrition Examination Survey, U.S., 1999–2014, were analyzed. For each of the eight cardiometabolic variables, borderline-risk and high-risk were considered unhealthy, and being unhealthy on any variable was considered “unhealthy overall” in terms of cardiometabolic risk. Area under the curve and *R* ^2^ were used to compare BMI percentile and AHtR for accuracy in predicting risk.

**Results:**

Female AHtR ≥ 0.19 and BMI percentile ≥ 94 and male AHtR ≥ 0.16 and BMI percentile ≥ 64 predicted a probability of >0.7 being unhealthy overall. AHtR predicted overall risk and unhealthy levels of six variables more accurately than BMI percentile. Significant differences were overall risk (*χ* ^2^ = 4.18; *p*=0.041), total cholesterol (*χ* ^2^ = 8.68; *p*=0.003), glycated hemoglobin (*χ* ^2^ = 5.24; *p*=0.022), and systolic pressure (*χ* ^2^ = 5.10; *p*=0.024). AHtR had higher accuracy in predicting high-density cholesterol, fasting glucose, glycated hemoglobin, and systolic/diastolic pressures plus higher specificity in predicting all variables except triglycerides. BMI percentile had higher sensitivity for all variables. Sensitivity and accuracy were higher for males. No significant race/ethnicity differences were observed.

**Conclusions:**

Without needing adjustment for age and weight, AHtR can predict some cardiometabolic risk factors of adolescents, especially of males, more accurately than BMI percentile, thus facilitating population risk estimation and early interventions. Further research is required to validate these findings in younger children.

## 1. Introduction

Child and adolescent overweight and obesity significantly increase the risk of premature mortality, morbidity (e.g., diabetes, asthma, and hypertension), and related outcomes [1]. Further, obesity tracks from childhood to adulthood more strongly than any other risk factor [2]. Risk factor clustering, both biological and behavioral, is the greatest predictor of accelerated atherosclerotic processes; such clustering during childhood and adolescence can persist into adulthood [3]. Although exact criteria are unclear, the ability to detect clustering of obesity and cardiometabolic risk factors in childhood and adolescence is important for early initiation of behavioral and medical interventions [4–6], which may include simple and inexpensive lifestyle modifications, such as increased daily sleep [7].

Unfortunately, self-reported anthropometric data from adolescents are often inaccurate [8]. In the past, this presented a meaningful problem, since these self-report risk factor data for a wide variety of potentially preventable chronic health issues were insufficient to effectively inform public health interventions. Thus, there was a need to identify a measure of obesity and overweight that is accurate, simple, and cost-effective [9]. BMI has emerged as the most commonly measured parameter, worldwide, for objectively assessing general obesity plus potential-related disease risk at all ages and establishing treatment, research, and evaluation priorities. Research has revealed that BMI is correlated with fat mass and percentage of body fat at 0.90 and 0.69, respectively, so it is highly correlated with waist circumference and moderately correlated with other measures of adiposity. Thus, BMI can be used to predict adiposity-related cardiometabolic risk in situations in which a high level of precision is not required [10]. However, there are several known barriers to the use of BMI. Calculation and plotting of child and adolescent BMI have been identified by some school-based healthcare professionals as prohibitive [11], especially on a large scale, due to the difficulty of the numeric conversions and time required to perform the calculations. In pediatric primary care, some physicians have expressed concern that parents will not understand the BMI measure or even that it may be misleading for certain body types [12], and a survey of the American Academy of Pediatrics fellowship found that pediatric primary care physicians, too, are less likely to calculate BMI in the absence of an assistive tool [13]. Some concurrent research has attempted to mitigate these barriers, for example, by using color-coded BMI charts to improve metric comprehension among parents [14]. Researchers from the World Health Organization recently acknowledged some issues with BMI, but noted that in absence of an alternative viable standard, BMI-for-age is currently the sole means used to identify individuals who are overweight or obese [15].

Alternative but far less prevalent measures include waist circumference and waist-to-height ratio, which are strong predictors of cardiometabolic risk in children [16], likely because these measure visceral fat (central obesity), which releases fatty acids, hormones, and inflammatory agents, ultimately increase cardiometabolic risk. Although uncommon in epidemiological studies, mid-upper-arm circumference of pediatric populations is included in some large-scale surveys, such as National Health and Nutrition Examination Survey (NHANES), United States. Mechanisms of measurement including the Quaker arm circumference measuring stick (QUAC-stick), which was introduced in the 1960s to measure children's height at 1–9 years in order to indicate nutritional status and facilitate comparisons between countries [17]. Although the ratio of muscle mass to fat mass in mid-upper-arm circumference varies by sex and age [18], children's mid-upper-arm circumference, concurrent with triceps skinfold thickness, has been used for community-wide nutritional assessment [19]. Mid-upper-arm circumference is commonly used to quickly identify moderate to severe acute undernutrition among 6–60 month olds in resource-limited settings [20]. Moreover, in pediatric emergencies, mid-upper-arm circumference is widely used as a proxy of body weight when adjusting ventilator/equipment settings and fluid/medication doses [20, 21].

While mid-upper-arm circumference-for-age reference tables were recently developed for 1–20 year olds US population [22], little research on use of mid-upper-arm circumference for determining overweight and obesity in children and adolescents exists [23–25]. Although mid-upper-arm circumference for height reference charts are available for 0.5–10 year olds [26], only one study has utilized mid-upper-arm circumference-to-height ratio (hereafter referred to as arm-to-height ratio or AHtR) to evaluate overweight and obesity in children [25]. Further, no studies have examined the potential of mid-upper-arm circumference or AHtR to predict cardiometabolic risk of children/adolescents. Given the relative simplicity of producing these measurements compared to BMI calculation, there is a potential value in validating AHtR as an alternative to BMI in time- or resource-limited environments, or even for pediatric patients whose parents have low numeric literacy. As a proof of concept, this study used pooled NHANES data across 8 cycles to (a) determine the suitability of utilizing AHtR to predict adolescents' cardiometabolic risk and (b) compare AHtR's predictive accuracy with corresponding BMI parameters.

## 2. Materials and Methods

### 2.1. Study Setting and Dates

The analyses were based on pooled, publicly available NHANES data collected from 1999 to 2000 through 2013-2014 (8 cycles), which covered a nationally representative sample from 15 US counties. NHANES uniquely combines demographic, socioeconomic, and health-related interview information with physical examination and laboratory data.

### 2.2. Participants

In each two-year cycle, NHANES surveyed approximately 5,000 noninstitutionalized persons, using complex, multistage, stratified, clustered, and random sampling designs that oversampled Hispanics and non-Hispanic Blacks. Certain NHANES variables are limited to ages 12 and above, while some measures are obtained from only a subsample (e.g., morning fasting sample). Therefore, the age range in this study was confined to adolescence (ages 12–18) and excluded pregnant females. Individuals aged 12–18 years were included only if they had valid height records, at least one additional anthropometric variable required for calculating BMI (i.e., weight) and AHtR (i.e., mid-upper-arm circumference) plus at least one of eight cardiometabolic variables. A total of 12,269 adolescents met these standards and were included; 5,005 records contained all eight variables. Between 1999 and 2014, unweighted response rates for adolescents ranged from 76% to 89% in the interviewed sample and 74%–87% in the examined sample.

#### 2.3.1. Demographic Variables

Age, gender, race/ethnicity, and antihypertensive, lipid-lowering, and antidiabetic medication use were self-reported.

#### 2.3.2. Anthropometric Variables

BMI and AHtR were calculated using height, weight, and mid-upper-arm circumference measurements from NHANES physicals. Using anthropometry protocols, mid-upper-arm circumference was taken on participants aged two months and older at the right upper arm mid-point mark. The examiner faced the right side of the upright standing participant whose shoulders were relaxed with arms hanging loosely without muscle flexing. Measurement was taken to the nearest 0.1 cm using a measuring tape, ensuring it did not compress the skin yet fit snugly. Mid-upper-arm circumference was divided by height to obtain “unitless” AHtR.

BMI percentiles were computed utilizing a CDC-provided SAS syntax for *z*-scores and percentiles based on sex and age according to 2000 CDC growth charts [27]. For participants aged 2–19, NHANES reported age in months before 2011-2012 and, thereafter, in years [28]. If age in months was unavailable, age in years was multiplied by 12, and then 6 was added, to prevent upwards biased *z*-scores; prior studies yielded negligible discrepancies with this approach [29]. Per established standards, healthy-weight was defined as BMI < 85^th^ percentile, overweight as ≥85^th^ to <95^th^ percentile, obesity as ≥ 95^th^ percentile to <120% of the 95^th^ percentile, and severe obesity as ≥120% of the 95^th^ percentile or a BMI > 35, regardless of age [30].

#### 2.3.3. Cardiometabolic Variables

Standard cutoffs in the Expert Panel Report 2012 of the National Heart, Lung, and Blood Institute were utilized for high-density lipoprotein cholesterol, low-density lipoprotein cholesterol, triglycerides, total cholesterol, and fasting plasma glucose; systolic and diastolic blood pressures were categorized based on age- and height-specific percentiles [2]. American Academy of Pediatrics (AAP) and American Diabetes Association (ADA) standard cutoffs were utilized for glycated hemoglobin [31]. Accordingly, healthy ranges for measures were (1) high-density lipoprotein cholesterol >45 mg/dL; (2) low-density lipoprotein cholesterol <110 mg/dL; (3) triglycerides <90 mg/dL; (4) total cholesterol <170 mg/dL; (5) fasting plasma glucose <100 mg/dL; (6) glycated hemoglobin <5.7%; (7) systolic blood pressure <90^th^ percentile; and (8) diastolic blood pressure <90^th^ percentile. In 18 year olds, systolic blood pressure <120 mmHg and diastolic blood pressure <80 mmHg were considered healthy [32]. Borderline-risk and high-risk range measurements were considered unhealthy. Adolescents were categorized as healthy for “overall cardiometabolic risk” if their scores fell within the healthy range for all cardiometabolic variables. Being unhealthy on any one variable was considered unhealthy on “overall cardiometabolic risk.”

### 2.4. Statistical Analysis

To assess BMI and AHtR outliers, each variable was converted to a normal distribution based on the best Box-Cox transformation. *Z*-scores were calculated after adjusting for age and sex. One outlier was removed, and 66 participants reporting lipid-lowering, antidiabetic, or antihypertensive medication use were reclassified from healthy to unhealthy. Data were combined across the eight survey cycles with sample weights combined as per survey guidelines. Combined weights were calculated for participants whose data were collected in NHANES mobile examination centers; separate weights were applied for participants in the fasting subsample who provided measures of fasting plasma glucose, low-density lipoprotein cholesterol, and triglycerides.

Binary logistic regression was performed on each cardiometabolic risk outcome (0 = healthy; 1 = unhealthy) as predicted by BMI percentile or AHtR, including a main effect and interaction with sex and race/ethnicity. A receiver operating characteristic (ROC) curve was calculated for each model, with area under the curve (AUC) used to compare the ability of BMI and AHtR to accurately predict unhealthy cardiometabolic variable levels (i.e., borderline-risk and high-risk levels); an AUC of one indicates a perfect predictor and ≤0.5 indicates a worthless predictor [33]. Chi-square tests were used to evaluate the statistical significance of AUC differences between AHtR and BMI percentiles after incorporating Bonferroni's adjustment for multiple comparisons. *R* ^2^ and Max-rescaled *R* ^2^ were also used as measures of model fit for comparison [34]. Another model tested overall cardiometabolic risk by combining all eight cardiometabolic measures to identify whether individuals were in the healthy range on all measures or in the unhealthy range (i.e., borderline-risk or high-risk) on any of the eight measures.

**Figure 1:**
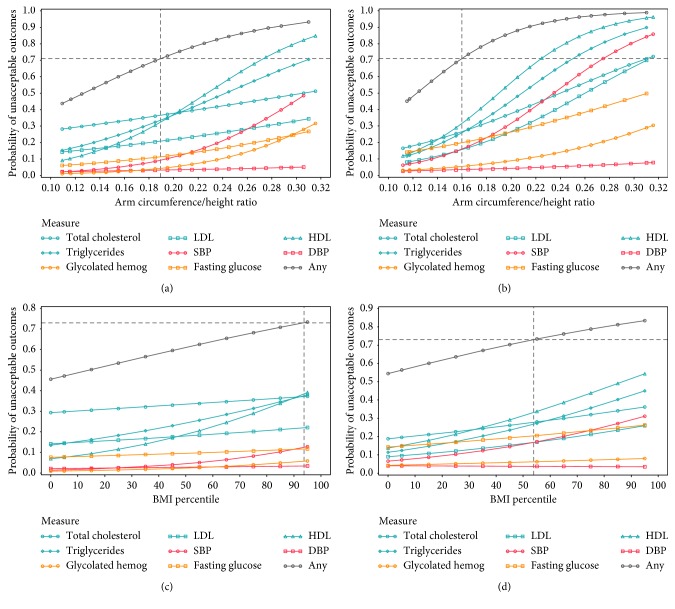
Changes in general obesity measures and corresponding cardiometabolic risk probabilities, ages 12–18 by sex, *N* = 12,268. (a) Probability of unacceptable outcomes, for females, (b) probability of unacceptable outcomes, for males, (c) BMI percentile and unhealthy level of each variable, for females, and (d) BMI percentile and unhealthy level of each variable, for females. CR = cardiometabolic risk; TC = total cholesterol; LDL-C = low-density lipoprotein cholesterol; HDL-C = high-density lipoprotein cholesterol; TG = triglycerides; SBP = systolic blood pressure; DBP = diastolic blood pressure; HbA1c = glycated hemoglobin; FPG = fasting plasma glucose; “Any” = unhealthy level on any of the eight CR variables (indicates “overall” CR). Unhealthy level of each CR variable includes both borderline-risk and high-risk levels, defined according to National Heart, Lung, and Blood Institute Expert Panel Report 2012, American Academy of Pediatrics, and American Diabetes Association. Arm-to-height ratio ≥ 0.19 in females and ≥ 0.16 in males regardless of age were identified as the best CR cutoffs, indicating ≥0.71 probability of having unhealthy level in any of the eight CR variables. BMI ≥ 94^th^ percentile in females and ≥64^th^ percentile in males at a given age were identified as the best CR cutoffs, indicating ≥0.73 probability of having unhealthy level in any of the eight CR variables.

Youden's Index, *J* (sensitivity + specificity − 100), identified the optimum age-invariant cutoff of AHtR separately for males and females (Figure 1), which best classified adolescents as healthy/unhealthy on overall cardiometabolic risk. Youden's Index is commonly used as a summary measure of the ROC curve because it can identify the maximum potential effectiveness of a biomarker [35]. For comparison, cardiometabolic cutoffs were identified for age-dependent BMI percentiles, separately for males and females, utilizing a similar approach. Based on these AHtR and BMI percentile cutoffs, participants were classified as high/low risk. For comparison with the current standard, individual risk level (high/low) was also determined based on whether the individual was above or below the conventional 85^th^ percentile of BMI (i.e., overweight).

SAS 9.4 SURVEYFREQ was used to calculate weighted frequencies based on survey weights, accounting for NHANES design effects of clustering and stratification with percentages based on weighted frequencies. Frequency and percentages for individuals aged 12–18 with healthy and unhealthy levels of each cardiometabolic variable within each BMI and AHtR level were cross-tabulated. Analyses included all adolescents plus males and females separately.

Logistic regression was repeated with each binary anthropometric indicator (high/low risk) to predict binary cardiometabolic outcomes (healthy/unhealthy). Altogether and separately for sex and race/ethnicity, classification was characterized and compared using sensitivity, specificity, positive predictive value, negative predictive value, accuracy ((true positives + true negatives)%), and *J*-statistic.

## 3. Results

Among 12,268 adolescents with calculated BMI, 62.7% (females 62.4%, males 63.0%; Hispanics 59.0%, Blacks 60.8%, Whites 68.1%, and other races 69.7%), were below the 85^th^ percentile of the reference population reported by the CDC in 2000 [27]. Further, 16.43%, 12.71%, and 8.18% met criteria for overweight, obesity, and severe obesity, respectively.  Table 1 provides AUC, *R* ^2^, and Max-rescaled *R* ^2^ as evaluation metrics for classifying cardiometabolic variables (healthy/unhealthy levels) based on AHtR and BMI percentiles. For all three parameters, AHtR predicted overall cardiometabolic risk (i.e., unhealthy level of any cardiometabolic variable) and unhealthy levels of six cardiometabolic variables (total cholesterol, high-density lipoprotein cholesterol, low-density lipoprotein cholesterol, glycated hemoglobin, systolic blood pressure, and diastolic blood pressure) more accurately than BMI percentiles. Four AUC differences between AHtR and BMI percentile were significant: overall cardiometabolic risk (*χ* ^2^ = 4.18; *p*=0.041), total cholesterol (*χ* ^2^ = 8.68; *p*=0.003), glycated hemoglobin (*χ* ^2^ = 5.24; *p*=0.022), and systolic blood pressure (*χ* ^2^ = 5.10; *p*=0.024). However, only total cholesterol remained significant after adjustment for multiple comparisons (*p*=0.038). With triglycerides and fasting plasma glucose, *R* ^2^ and Max-rescaled *R* ^2^ were better for AHtR, but AUC was better for BMI percentile, with a statistically nonsignificant difference.

The effect of AHtR on cardiometabolic risk did not depend on age, i.e., the interaction was not statistically significant. The AHtR age-invariant cutoff was ≥0.19 for females and ≥0.16 for males, with a ≥0.71 probability of being unhealthy on any of the eight cardiometabolic variables (Figure 1). Among 12,140 adolescents with calculated AHtR, 54.88% were below cutoff (males 36.52%, females 74.23%; Hispanics 52.08%, Whites 58.91%, Blacks 53.01%, and other races 62.64%). Comparable cardiometabolic cutoffs of BMI percentiles were ≥94^th^ for females and ≥64^th^ for males, with a ≥0.73 probability of being unhealthy on any of the eight cardiometabolic variables. Among 12,268 adolescents with calculated BMI percentiles, 54.99% were below cutoff (males 34.01%, females 77.05%; Hispanics 52.84%, Whites 59.07%, Blacks 52.14%, and other races 63.05%). Figure 1 plots changes in each cardiometabolic variable and overall cardiometabolic risk against changes in AHtR and BMI percentile by sex. Compared to BMI percentile, greater AHtR slopes were observed, with more curve around the cutoff, especially in males.  Table 2 demonstrates weighted percentages of adolescents, in aggregate and by sex, with unhealthy levels of each cardiometabolic variable within each level of anthropometric variable. Of all cardiometabolic variables, unhealthy levels of lipid-panel variables (total cholesterol, high-density lipoprotein cholesterol, low-density lipoprotein cholesterol, and triglycerides) contributed most to the increased overall cardiometabolic risk.

Table 3 presents sensitivity, specificity, positive predictive value, negative predictive value, accuracy, and *J*-statistic for overall cardiometabolic risk classification (healthy/unhealthy), using each binary anthropometric indicator (high/low risk), in all adolescents and separately by sex. For cardiometabolic cutoffs of AHtR and BMI percentile, male sensitivity and accuracy were higher, while female specificity was higher. For all adolescents and separately for males, sensitivity, negative predictive value, accuracy, and *J*-statistic were highest with use of the age-invariant AHtR cutoff. For females, sensitivity, negative predictive value, accuracy, and *J*-statistic were highest with the use of BMI 85^th^ percentile, while specificity and positive predictive value were highest with BMI 94^th^ percentile (cardiometabolic cutoff). For all individuals and separately for males, specificity and positive predictive values were highest with the use of BMI 85^th^ percentile.

Table 3 also demonstrates the sensitivity, specificity, positive predictive value, negative predictive value, accuracy, and *J*-statistic for classification of each binary cardiometabolic variable in all adolescents, using each binary anthropometric indicator. While AHtR had the highest accuracy for high-density lipoprotein cholesterol, fasting plasma glucose, glycated hemoglobin, systolic blood pressure, and diastolic blood pressure as well as highest specificity for all cardiometabolic variables except for triglycerides, BMI 85^th^ percentile had the highest accuracy for total cholesterol, low-density lipoprotein cholesterol, and triglycerides. The cardiometabolic cutoff of BMI percentile had the highest sensitivity for all cardiometabolic variables. Appendices 1 and 2 provide corresponding results for females and males, respectively, by race/ethnicity.

## 4. Discussion

The current study comparing AHtR to BMI percentile is a “proof of concept” for AHtR's ability to predict adolescent cardiometabolic risk. In a prior Chinese study, AHtR ≥ 0.15 in both sexes was associated with elevated BMI, with sensitivities of 86.0% and 85.4% plus specificities of 91.5% and 87.8% for males and females, respectively [25]. In the current study, AHtR ≥ 0.19 in males and AHtR ≥ 0.16 in females predicted a ≥0.71 probability of being unhealthy on overall cardiometabolic risk, with higher sensitivity and classification accuracy for males. BMI ≥ 94^th^ percentile in females and BMI ≥ 64^th^ percentile in males predicted comparable risk. The observed sex difference is in agreement with the prior nationally representative studies. May et al. found that adolescent males were more likely than females to have one (36% vs 28%), two (10% vs 7%), or more than two (4% vs 0.8%) cardiometabolic risk factors, whereas a consistent dose-response increase in the prevalence of cardiometabolic risk factors was observed by weight categories [36]. A sex difference was also observed consistently across racial/ethnic groups. For example, Hispanic/Latino males had a greater prevalence of diabetes (21% vs 12%) and hypertension (9% vs 3%) and were more likely to have prediabetes (OR = 2) than their female counterparts [37]. Although the current study did not find considerable difference by race/ethnicity, cross-national validation of these results is warranted.

One meaningful impetus for this study was the research literature suggesting that BMI is not a perfect measure of general obesity [38]—specifically, the findings that healthcare providers indicated that the complexity of BMI was a barrier to its regular use [11–13]. BMI involves commonly understood body weight and height, but is somewhat complicated to calculate. For children and adolescents, assessors interpret age-sex specific BMI percentile charts, requiring additional numeric skills. Importantly, BMI has moved to the forefront of recommended metrics due to its accuracy, simplicity, and low cost [7]. Thus, the finding that AHtR performed similarly to BMI in calculating cardiometabolic risk factors in adolescents in this sample may suggest its potential use as a complementary measure of general obesity in situations where one or more barriers to using BMI are present. In contrast, many other measures of total adiposity, such as dual-energy x-ray absorptiometry, are more difficult than BMI to obtain in ordinary survey and clinical settings, and most importantly, in homes, precluding self-monitoring. Further, measuring central obesity using waist circumference per guidelines by minimizing the effects of clothing, respiration, and stomach content and palpating iliac crest and costal margin accurately may prove difficult [39]. It may also raise ethical and cultural concerns due to intrusiveness, especially in the context of surveillance programs.

Compared to BMI percentile, use of AHtR for cardiometabolic risk prediction may be advantageous in some situations because it is (1) not age-dependent—does not require percentile charts; (2) not weight-dependent—does not require weighing scales, does not require daily scale calibration [40], and is not sensitive to short-term weight fluctuations; (3) a ratio of two lengths, hence, interpretable without unit conversion; and (4) easier to calculate—requires less time and effort. As a measure, AHtR also has limitations. Its prediction accuracy is lower than BMI for females. Additionally, as with other circumference measures [41], mid-upper-arm circumference has no uniformly accepted measurement approach and is susceptible to measurement errors. While prior studies revealed significant correlations between mid-upper-arm circumference and height [25, 42], this study considered adolescents' height as an empirically established alternative to age for evaluating obesity [16].

AHtR also has advantages over mid-upper-arm circumference. For US children and adolescents aged 1–20 years, mid-upper-arm circumference-for-age reference charts, including arm muscle area (AMA) and arm fat area (AFA), are available [22]. However, height status had complex independent relationships with arm measures, irrespective of the ranking by age and sex [22]. Mei et al. indicated that it is difficult to assess a child's age in some circumstances [26]. Even if the age is known, use of age-based mid-upper-arm circumference percentile charts may be complicated, due to lack of resources, knowledge, and skills. AHtR may be a good alternative in such circumstances. Recent studies demonstrated a steady increase of adolescent mid-upper-arm circumference with age, although increases in mid-upper-arm circumference components had age- and sex-related differences [22]. Upper-arm fat mass gains were slower in males from 12 to 15 years of age, compared to more-intense increases among females throughout adolescence. However, upper-arm muscle mass had sharper gains with age in males than in females [22]. Accordingly, the identification of sex-specific AHtR cutoffs in this study was consistent with prior research.

As children with overweight or obesity are more likely to have unhealthy levels of cardiometabolic risk [43, 44], inclusion of varying degrees of obesity may increase the accuracy of cardiometabolic risk prediction [43]. Numerous studies have evaluated the association between obesity and other biological risk factors [2], with some suggesting that BMI is imperfect for predicting cardiometabolic risk, considering metabolically obese healthy-weight subjects [45] and metabolically healthy subjects with obesity [46]. Conversely, some researchers have argued that BMI performed better than body fat mass in predicting cardiometabolic risk [47]. Furthermore, central obesity had a stronger association with cardiometabolic risk in children than general obesity [16]. While predictive accuracy is a major concern in determining the suitability of anthropometric indicators for cardiometabolic risk prediction in survey and clinical settings, low-cost and simplicity also can be crucial. This study revealed that, compared to BMI percentile, AHtR classified overall cardiometabolic risk and several cardiometabolic variables better in males. These results were consistent with prior studies which revealed that mid-upper-arm circumference correlates more with systolic blood pressure than with diastolic blood pressure [48], although empirical evidence is unavailable for other risk factors.

This study had several overarching limitations. Children under 12 years were excluded because some cardiometabolic variables were unavailable for them, limiting the conclusions that can be drawn about the appropriateness of AHtR for that population. This study did not compare measures of central obesity or body fat mass with AHtR and BMI percentile, because the focus was general obesity; however, numerous studies have compared those measures with BMI percentile [43, 44]. Also, this study did not analyze correlations of AHtR with other anthropometric parameters and age, as it was designed to identify a meaningful sex-specific AHtR cutoff that predicts a higher cardiometabolic risk, a concept which is similar to widely used cutoff value 0.5 for waist-to-height ratio. The optimal AHtR and BMI percentile cutoffs for the cardiometabolic risk assessment were not cross-validated in an independent sample. Due to the cross-sectional design, this study was unable to examine changes in anthropometric variables and cardiometabolic risks over the 16-year-period, establish causality between obesity and cardiometabolic risk, or determine associated morbidity and mortality. In addition, NHANES deliberately oversampled Black and Hispanic adolescents. Only Whites, Blacks, and Hispanics were included in the analysis because the number of other racial/ethnic group participants was insufficient. Underweight adolescents (BMI < 5^th^ percentile; *n*=726) were merged with healthy range for BMI, considering that the focus of this study was obesity. Further, for multifactorial conditions, such as obesity and cardiometabolic abnormalities, acceptance of any given parameter solely as “unhealthy” may not describe the whole picture, as there can be several interparameter relations. Similarly, the coexistence of multiple cardiometabolic risk factors should be treated as worse than having just one, and ideally, different levels of risk (e.g., one risk factor and two risk factors) should be assessed. Finally, some individuals had missing values for one or more cardiometabolic or anthropometric variables.

Behavioral interventions that effectively reduce the weight of children with overweight demonstrate gradually smaller effects at greater obesity levels [5], with interventions displaying an inverse correlation between effectiveness and age [6]. Furthermore, children with obesity have greater risk of developing cardiometabolic abnormalities early in life [4]. This study indicated that, as a simple and low-cost measure of general obesity, AHtR can reasonably predict cardiometabolic risk in adolescents and has a higher predictive accuracy than BMI percentile in males, thus allowing for early interventions. This may be especially pertinent in contexts where one or more barriers to BMI computation exist. Further research is required to validate these findings in younger children. Attention should be paid to include AHtR (or mid-upper-arm circumference in addition to height) in adolescent anthropometric surveys, school-based surveillance programs, and clinical evaluations, whenever cardiometabolic risk prediction is considered worthwhile, but invasive procedures are not feasible or permissible.

## Figures and Tables

**Table 1:** Evaluation metrics for ability of general obesity measures to predict cardiometabolic risk, ages 12–18, *N* = 12,268.

Cardiometabolic risk variables	General obesity measures						AUC difference between arm-to-height ratio and BMI percentile
	Arm-to-height ratio (sex-specific)			BMI percentile (age- and sex-specific)			
	*R* ^2^	Max-rescaled *R* ^2^	AUC	*R* ^2^	Max-rescaled *R* ^2^	AUC	
Overall risk	0.058	0.083	0.647	0.044	0.063	0.638	*χ* ^2^ = 4.18; *p*=0.041
TC	0.018	0.025	0.585	0.012	0.017	0.577	*χ* ^2^ = 8.68; *p*=0.003
LDL-C	0.021	0.033	0.607	0.014	0.022	0.595	*χ* ^2^ = 3.62; *p*=0.057
HDL-C	0.098	0.136	0.691	0.088	0.122	0.689	*χ* ^2^ = 1.18; *p*=0.278
TG	0.053	0.075	0.646	0.049	0.069	0.648	*χ* ^2^ = 0.24; *p*=0.621
SBP	0.082	0.143	0.728	0.072	0.126	0.722	*χ* ^2^ = 5.10; *p*=0.024
DBP	0.001	0.004	0.545	0.001	0.003	0.538	*χ* ^2^ = 0.35; *p*=0.556
HbA1C	0.016	0.048	0.646	0.010	0.029	0.634	*χ* ^2^ = 5.24; *p*=0.022
FPG	0.034	0.058	0.640	0.031	0.052	0.642	*χ* ^2^ = 0.70; *p*=0.403

AUC = area under the curve; TC = total cholesterol; LDL-C = low-density lipoprotein cholesterol; HDL-C = high-density lipoprotein cholesterol; TG = triglycerides; SBP = systolic blood pressure; DBP = diastolic blood pressure; HbA1c = glycated hemoglobin; FPG = fasting plasma glucose; unhealthy level of each cardiometabolic risk variable includes both borderline-risk and high-risk levels, defined according to National Heart, Lung, and Blood Institute Expert Panel Report 2012, American Academy of Pediatrics, and American Diabetes Association. Overall risk indicates unhealthy level on any of the eight CR variables.

**Table 2:** Weighted percent^*∗*^ for unhealthy levels of cardiometabolic risk variables within levels of general obesity measures, *N* = 12,268.

Gender	Predictor	Categories	Overall CR	TC	LDL-C	HDL-C	TG	SBP	DBP	HbA1C	FPG
All adolescents	Conventional cutoffs of BMI percentile	Healthy	63.7	27.6	15.6	25.6	24.8	9.3	3.0	3.5	14.5
		Overweight	75.2	34.6	23.2	43.6	36.5	17.0	2.8	3.5	16.9
		Obese	84.8	40.4	26.7	56.1	49.3	22.9	5.3	4.5	21.9
		Severe obese	89.3	41.7	35.2	71.3	59.8	31.7	4.4	12.9	30.2
	CR cutoffs of BMI percentile	Low risk	61.7	29.9	17.1	23.8	24.8	7.5	3.0	3.2	11.7
		High risk	80.8	33.2	22.7	50.5	41.5	21.9	3.7	5.6	24.1
	CR cutoffs of arm-to-height ratio	Low risk	61.5	28.8	16.0	24.2	25.1	7.4	2.9	3.3	13.1
		High risk	81.0	34.7	24.2	50.2	41.1	22.3	3.9	5.6	22.2

Females	Conventional cutoffs of BMI percentile	Healthy	59.4	32.6	17.2	19.6	24.9	4.5	3.0	2.6	9.2
		Overweight	67.3	35.7	21.9	35.9	33.3	8.7	2.3	3.4	8.0
		Obese	80.1	41.7	25.1	45.0	42.0	10.0	5.1	4.8	13.8
		Severe obese	86.2	39.5	30.1	63.3	49.8	23.5	3.0	10.5	20.4
	CR cutoffs of BMI percentile	Low risk	60.8	33.2	18.5	22.6	26.2	5.3	2.8	2.8	9.3
		High risk	80.6	40.3	25.2	49.8	43.8	14.3	4.3	6.5	15.1
	CR cutoffs of arm-to-height ratio	Low risk	60.7	32.8	18.3	22.2	26.8	5.2	2.9	2.7	9.7
		High risk	78.7	40.6	25.2	47.5	39.7	13.3	3.8	6.0	13.0

Males	Conventional cutoffs of BMI percentile	Healthy	67.9	22.9	14.0	31.4	24.8	14.0	3.0	4.4	19.7
		Overweight	82.8	33.5	24.5	50.7	39.6	24.8	3.3	3.5	25.3
		Obese	89.1	39.3	28.3	65.9	56.0	33.9	5.5	4.3	29.4
		Severe obese	91.9	43.7	39.6	78.2	68.1	38.8	5.5	14.9	38.4
	CR cutoffs of BMI percentile	Low risk	63.5	22.6	14.1	26.3	21.9	12.3	3.5	4.3	16.6
		High risk	80.9	31.1	22.0	50.7	40.8	24.1	3.6	5.4	26.7
	CR cutoffs of arm-to-height ratio	Low risk	63.2	21.1	11.8	28.0	22.1	11.5	3.0	4.2	19.3
		High risk	81.8	32.5	23.9	51.1	41.6	25.4	3.9	5.5	25.4

CR = cardiometabolic risk; TC = total cholesterol; LDL-C = low-density lipoprotein cholesterol; HDL-C = high-density lipoprotein cholesterol; TG = triglycerides; SBP = systolic blood pressure; DBP = diastolic blood pressure; HbA1c = glycated hemoglobin; FPG = fasting plasma glucose. Overall CR indicates unhealthy level on any of the eight CR variables. Unhealthy level of each CR variable includes both borderline-risk and high-risk levels, defined according to National Heart, Lung, and Blood Institute Expert Panel Report 2012, American Academy of Pediatrics, and American Diabetes Association. Conventionally, BMI < 85^th^ percentile was considered healthy; ≥85^th^ to <95^th^ percentile, overweight; ≥95^th^ percentile to <120% of 95^th^ percentile, obese; ≥120% of 95^th^ percentile or BMI > 35 regardless of age was severe obese. For CR, BMI ≥ 94^th^ percentile in females and BMI ≥ 64^th^ percentile in males, at a given age, as well as arm-to-height ≥ 0.19 in females and arm-to-height ≥ 0.16 in males, regardless of age, were considered high-risk. ^*∗*^Sample weights were utilized across the eight survey cycles as per survey guidelines. Combined weights were calculated for participants whose data were collected in mobile examination centers; separate weights were applied for participants in the fasting subsample who provided measures of FPG, LDL-C, and TG.

**Table 3:** Characteristics of overall and specific cardiometabolic risk classifications, using measures of general obesity, ages 12–18, *N* = 12,268.

CR	Predictor	Sensitivity	Specificity	PPV	NPV	True +	False +	True −	False −	Total	Accuracy (%)	*J*
Overall all adolescents	BMI ≥ 85^th^ percentile	42.78	77.73	82.16	36.17	1511	328	1145	2021	5005	53.07	20.51
	CR cutoffs, BMI percentile	51.08	70.67	80.68	37.59	1774	432	1041	1758	5005	56.24	21.75
	CR cutoffs, arm-to-height	52.01	70.67	80.96	38.05	1837	432	1041	1695	5005	57.50	22.68

Overall female	BMI ≥ 85^th^ percentile	43.88	74.85	76.97	41.05	685	205	610	876	2376	54.50	18.73
	CR cutoffs, BMI percentile	27.80	88.22	81.89	38.95	434	96	719	1127	2376	48.53	16.02
	CR cutoffs, arm-to-height	30.17	84.05	78.37	38.59	471	130	685	1090	2376	48.65	14.22

Overall male	BMI ≥ 85^th^ percentile	41.91	81.31	87.04	31.85	826	123	535	1145	2629	51.77	23.22
	CR cutoffs, BMI percentile	67.99	48.94	79.95	33.79	1340	336	322	631	2629	63.22	16.93
	CR cutoffs, arm-to-height	69.30	54.1	81.89	37.04	1366	302	356	605	2629	65.50	23.40

TC	BMI ≥ 85^th^ percentile	45.33	66.02	39.15	71.46	1646	2558	4969	1985	11158	59.28	11.35
	CR cutoffs, BMI percentile	45.47	61.68	36.41	70.10	1651	2884	4643	1980	11158	56.41	7.15
	CR cutoffs, arm-to-height	41.86	66.60	37.68	70.37	1520	2514	5013	2111	11158	58.55	8.46

LDL-C	BMI ≥ 85^th^ percentile	49.85	66.59	26.69	84.47	505	1387	2764	508	5164	63.30	16.44
	CR cutoffs, BMI percentile	49.85	62.25	24.37	83.57	505	1567	2584	508	5164	59.82	12.10
	CR cutoffs, arm-to-height	46.69	66.59	25.43	83.66	473	1387	2764	540	5164	62.68	13.28

HDL-C	BMI ≥ 85^th^ percentile	55.62	71.54	50.12	75.82	2107	2097	5272	1681	11157	66.14	27.17
	CR cutoffs, BMI percentile	59.64	69.13	49.82	76.91	2259	2275	5094	1529	11157	65.90	28.76
	CR cutoffs, arm-to-height	54.80	73.43	51.46	75.97	2076	1958	5411	1712	11157	67.11	28.23

TG	BMI ≥ 85^th^ percentile	53.16	70.74	45.12	76.95	882	1073	2594	777	5326	65.26	23.90
	CR cutoffs, BMI percentile	54.97	66.65	42.72	76.59	912	1223	2444	747	5326	63.01	21.62
	CR cutoffs, arm-to-height	50.63	70.55	43.75	75.95	840	1080	2587	819	5326	64.34	21.18

SBP	BMI ≥ 85^th^ percentile	58.18	66.48	23.48	89.99	1046	3409	6760	752	11967	65.23	24.65
	CR cutoffs, BMI percentile	67.19	64.54	25.09	91.75	1208	3606	6563	590	11967	64.94	31.73
	CR cutoffs, arm-to-height	62.24	69.01	26.21	91.18	1119	3151	7018	679	11967	68.00	31.25

DBP	BMI ≥ 85^th^ percentile	39.49	62.95	3.53	96.81	156	4266	7247	239	11908	62.17	2.44
	CR cutoffs, BMI percentile	43.80	60.04	3.62	96.89	173	4601	6912	222	11908	59.50	3.83
	CR cutoffs, arm-to-height	41.77	64.65	3.90	97.00	165	4070	7443	230	11908	63.89	6.42

HbA1C	BMI ≥ 85^th^ percentile	51.32	63.22	7.30	95.83	310	3934	6762	294	11300	62.58	14.54
	CR cutoffs, BMI percentile	57.45	60.42	7.58	96.18	347	4233	6463	257	11300	60.27	17.87
	CR cutoffs, arm-to-height	51.82	64.86	7.69	95.97	313	3759	6937	291	11300	64.16	16.68

FPG	BMI ≥ 85^th^ percentile	45.45	65.18	20.54	85.79	405	1567	2933	486	5391	61.92	10.63
	CR cutoffs, BMI percentile	56.00	63.31	23.21	87.90	499	1651	2849	392	5391	62.10	19.32
	CR cutoffs, arm-to-height	49.16	66.76	22.65	86.90	438	1496	3004	453	5391	63.85	15.91

CR = cardiometabolic risk; TC = total cholesterol; LDL-C = low-density lipoprotein cholesterol; HDL-C = high-density lipoprotein cholesterol; TG = triglycerides; SBP = systolic blood pressure; DBP = diastolic blood pressure; HbA1c = glycated hemoglobin; FPG = fasting plasma glucose. Unhealthy level of each CR variable includes both borderline-risk and high-risk levels, defined according to National Heart, Lung, and Blood Institute Expert Panel Report 2012, American Academy of Pediatrics, and American Diabetes Association. PPV = positive predictive value; NPV = negative predictive value; true + = number of true positives; false + = number of false positives; true − = number of true negatives; false − = number of false negatives; accuracy = percent of (true positives + true negatives); *J* = sensitivity + specificity − 100 (i.e., Youden's Index). Conventionally, BMI ≥ 85^th^ percentile (overweight) was considered high risk. For CR, BMI ≥ 94^th^ percentile in females and BMI ≥ 64^th^ percentile in males, at a given age, as well as arm-to-height ≥ 0.19 in females and arm-to-height ≥ 0.16 in males, regardless of age, were considered high risk.

## Supplementary Materials

Supplementary Materials Appendix 1 provides the sensitivity, specificity, positive predictive value, negative predictive value, accuracy, and *J*-statistic for classification of each binary cardiometabolic variable for females by race/ethnicity, using each binary anthropometric indicator. Appendix 2 provides corresponding data for males.
