# Supplementary material for: Arm Circumference-to-Height Ratio as a Situational Alternative to BMI Percentile in Assessing Obesity and Cardiometabolic Risk in Adolescents
Source: J Obes. 2018 Sep 6;2018:7456461. doi: 10.1155/2018/7456461 (PMC6146674; doi:10.1155/2018/7456461)
Supplement: Supplementary Materials — Appendix 1 provides the sensitivity, specificity, positive predictive value, negative predictive value, accuracy, and J-statistic for classification of each binary cardiometabolic variable for females by race/ethnicity, using each binary anthropometric indicator. Appendix 2 provides corresponding data for males. [file 7456461.f1.pdf]

Appendix-1: Statistical Measures for Classification of Each Binary Cardiometabolic Risk Variable, Using Each Binary Measure of General Obesity, Reference Table for Females of Ages 12-18 by Race and Ethnicity, N=5,979

Race	CR	Predictor	Sensi	Speci	PPV	NPV	Tr+	Fal+	Tr-	Fal-	Total	Accur	J
Females of All Races/Ethnicities	TC	BMI ≥ 85th Percentile	42.14	64.24	38.75	67.40	796	1258	2260	1093	5407	56.52%	6.38
		CR Cutoffs, BMI Percentile	25.99	79.36	40.35	66.63	491	726	2792	1398	5407	60.72%	5.36
		CR Cutoffs, Arm/Height	24.25	80.53	40.07	66.44	458	685	2833	1431	5407	60.87%	4.77
	LDL-C	BMI ≥ 85th Percentile	45.00	64.51	23.66	82.76	216	697	1267	264	2444	60.68%	9.51
		CR Cutoffs, BMI Percentile	27.71	79.38	24.72	81.79	133	405	1559	347	2444	69.23%	7.09
		CR Cutoffs, Arm/Height	24.79	80.45	23.66	81.40	119	384	1580	361	2444	69.52%	5.24
	HDL-C	BMI ≥ 85th Percentile	57.50	69.46	41.82	81.06	859	1195	2718	635	5407	66.15%	26.96
		CR Cutoffs, BMI Percentile	39.69	84.05	48.73	78.50	593	624	3289	901	5407	71.80%	23.75
		CR Cutoffs, Arm/Height	36.88	84.87	48.21	77.88	551	592	3321	943	5407	71.61%	21.75
	TG	BMI ≥ 85th Percentile	51.46	68.70	41.17	76.88	387	553	1214	365	2519	63.56%	20.17
		CR Cutoffs, BMI Percentile	34.31	83.31	46.65	74.87	258	295	1472	494	2519	68.68%	17.61
		CR Cutoffs, Arm/Height	30.98	83.98	45.16	74.09	233	283	1484	519	2519	68.16%	14.97
	SBP	BMI ≥ 85th Percentile	64.48	64.87	13.93	95.40	305	1885	3481	168	5839	64.84%	29.35
		CR Cutoffs, BMI Percentile	45.88	79.78	16.67	94.36	217	1085	4281	256	5839	77.03%	25.66
		CR Cutoffs, Arm/Height	44.19	81.05	17.05	94.28	209	1017	4349	264	5839	78.06%	25.23
	DBP	BMI ≥ 85th Percentile	43.60	62.73	3.44	97.34	75	2108	3548	97	5828	62.17%	6.33
		CR Cutoffs, BMI Percentile	29.07	77.92	3.85	97.31	50	1249	4407	122	5828	76.48%	6.99
		CR Cutoffs, Arm/Height	27.91	79.26	3.93	97.31	48	1173	4483	124	5828	77.75%	7.17
	HbA1C	BMI ≥ 85th Percentile	63.38	63.25	6.51	97.71	135	1938	3335	78	5486	63.25%	26.63
		CR Cutoffs, BMI Percentile	46.01	78.55	7.97	97.30	98	1131	4142	115	5486	77.29%	24.56
		CR Cutoffs, Arm/Height	44.13	79.90	8.15	97.25	94	1060	4213	119	5486	78.51%	24.03
	FPG	BMI ≥ 85th Percentile	46.99	64.15	13.21	91.24	125	821	1469	141	2556	62.36%	11.14
		CR Cutoffs, BMI Percentile	33.46	79.65	16.04	91.15	89	466	1824	177	2556	74.84%	13.11
		CR Cutoffs, Arm/Height	30.83	80.92	15.80	90.97	82	437	1853	184	2556	75.70%	11.74
Non-Hispanic White Females	Overall	BMI ≥ 85th Percentile	31.54	81.19	76.70	37.66	135	41	177	293	646	48.30%	12.73
		CR Cutoffs, BMI Percentile	19.16	91.28	81.19	36.51	82	19	199	346	646	43.50%	10.44
		CR Cutoffs, Arm/Height	24.07	87.16	78.63	36.89	103	28	190	325	646	45.36%	11.22
	TC	BMI ≥ 85th Percentile	36.06	72.21	41.61	67.28	181	254	660	321	1416	59.39%	8.27
		CR Cutoffs, BMI Percentile	20.72	85.23	43.51	66.19	104	135	779	398	1416	62.36%	5.95
		CR Cutoffs, Arm/Height	21.31	85.23	44.21	66.35	107	135	779	395	1416	62.57%	6.54
	LDL-C	BMI ≥ 85th Percentile	37.50	75.66	27.12	83.37	48	129	401	80	658	68.24%	13.16
		CR Cutoffs, BMI Percentile	21.09	85.85	26.47	81.83	27	75	455	101	658	73.25%	6.94
		CR Cutoffs, Arm/Height	19.53	85.85	25.00	81.54	25	75	455	103	658	72.95%	5.38
	HDL-C	BMI ≥ 85th Percentile	46.82	76.19	45.75	76.96	199	236	755	226	1416	67.37%	23.01
		CR Cutoffs, BMI Percentile	29.18	88.40	51.88	74.43	124	115	876	301	1416	70.62%	17.57
		CR Cutoffs, Arm/Height	28.00	87.59	49.17	73.94	119	123	868	306	1416	69.70%	15.59
	TG	BMI ≥ 85th Percentile	37.50	78.00	45.90	71.49	84	99	351	140	674	64.54%	15.50
		CR Cutoffs, BMI Percentile	22.77	88.00	48.57	69.60	51	54	396	173	674	66.32%	10.77
		CR Cutoffs, Arm/Height	21.88	87.78	47.12	69.30	49	55	395	175	674	65.88%	9.65
	SBP	BMI ≥ 85th Percentile	51.52	71.19	10.97	95.52	51	414	1023	48	1536	69.92%	22.71
		CR Cutoffs, BMI Percentile	35.35	84.90	13.89	95.02	35	217	1220	64	1536	81.71%	20.25
		CR Cutoffs, Arm/Height	35.35	84.76	13.78	95.01	35	219	1218	64	1536	81.58%	20.11
	DBP	BMI ≥ 85th Percentile	31.37	69.84	3.46	96.73	16	447	1035	35	1533	68.56%	1.21
		CR Cutoffs, BMI Percentile	25.49	83.94	5.18	97.04	13	238	1244	38	1533	82.00%	9.43
		CR Cutoffs, Arm/Height	23.53	83.81	4.76	96.96	12	240	1242	39	1533	81.80%	7.34
	HbA1C	BMI ≥ 85th Percentile	34.48	69.64	2.29	98.09	10	426	977	19	1432	68.92%	4.12
		CR Cutoffs, BMI Percentile	24.14	83.39	2.92	98.15	7	233	1170	22	1432	82.19%	7.53
		CR Cutoffs, Arm/Height	24.14	83.25	2.89	98.15	7	235	1168	22	1432	82.05%	7.39
	FPG	BMI ≥ 85th Percentile	31.34	73.69	11.54	90.74	21	161	451	46	679	69.51%	5.04
		CR Cutoffs, BMI Percentile	22.39	85.29	14.29	90.94	15	90	522	52	679	79.09%	7.68
		CR Cutoffs, Arm/Height	19.40	85.13	12.50	90.61	13	91	521	54	679	78.65%	4.53

Continued on the Next Page.

Race	CR	Predictor	Sensi	Speci	PPV	NPV	Tr+	Fal+	Tr-	Fal-	Total	Accur	J
Non-Hispanic Black Females	Overall	BMI ≥ 85th Percentile	53.09	63.98	71.67	44.28	215	85	151	190	641	57.10%	17.07
		CR Cutoffs, BMI Percentile	34.07	80.08	74.59	41.45	138	47	189	267	641	51.01%	14.16
		CR Cutoffs, Arm/Height	35.80	75.42	71.43	40.64	145	58	178	260	641	50.39%	11.23
	TC	BMI ≥ 85th Percentile	45.18	55.68	36.88	63.92	253	433	544	307	1537	51.85%	0.86
		CR Cutoffs, BMI Percentile	28.39	72.47	37.15	63.84	159	269	708	401	1537	56.41%	0.86
		CR Cutoffs, Arm/Height	25.54	74.92	36.86	63.71	143	245	732	417	1537	56.93%	0.46
	LDL-C	BMI ≥ 85th Percentile	51.68	54.05	24.44	79.55	77	238	280	72	667	53.52%	5.73
		CR Cutoffs, BMI Percentile	29.53	71.43	22.92	77.89	44	148	370	105	667	62.07%	0.96
		CR Cutoffs, Arm/Height	26.17	72.59	21.55	77.37	39	142	376	110	667	62.22%	-1.24
	HDL-C	BMI ≥ 85th Percentile	66.67	61.96	34.40	86.13	236	450	733	118	1537	63.04%	28.63
		CR Cutoffs, BMI Percentile	49.44	78.61	40.89	83.86	175	253	930	179	1537	71.89%	28.05
		CR Cutoffs, Arm/Height	46.05	80.98	42.01	83.38	163	225	958	191	1537	72.93%	27.03
	TG	BMI ≥ 85th Percentile	69.16	57.65	22.70	91.22	74	252	343	33	702	59.40%	26.81
		CR Cutoffs, BMI Percentile	50.47	75.80	27.27	89.48	54	144	451	53	702	71.94%	26.27
		CR Cutoffs, Arm/Height	46.73	77.31	27.03	88.97	50	135	460	57	702	72.65%	24.04
	SBP	BMI ≥ 85th Percentile	63.86	58.32	17.36	92.17	129	614	859	73	1675	58.99%	22.18
		CR Cutoffs, BMI Percentile	47.52	74.68	20.47	91.21	96	373	1100	106	1675	71.40%	22.20
		CR Cutoffs, Arm/Height	41.58	76.78	19.72	90.55	84	342	1131	118	1675	72.54%	18.37
	DBP	BMI ≥ 85th Percentile	56.06	56.23	5.00	96.89	37	703	903	29	1672	56.22%	12.29
		CR Cutoffs, BMI Percentile	31.82	72.17	4.49	96.26	21	447	1159	45	1672	70.57%	3.99
		CR Cutoffs, Arm/Height	31.82	74.84	4.94	96.39	21	404	1202	45	1672	73.15%	6.66
	HbA1C	BMI ≥ 85th Percentile	67.86	57.54	10.94	95.89	76	619	839	36	1570	58.28%	25.40
		CR Cutoffs, BMI Percentile	50.00	74.07	12.90	95.07	56	378	1080	56	1570	72.36%	24.07
		CR Cutoffs, Arm/Height	45.54	76.47	12.94	94.81	51	343	1115	61	1570	74.27%	22.01
	FPG	BMI ≥ 85th Percentile	64.41	55.62	11.52	94.57	38	292	366	21	717	56.35%	20.03
		CR Cutoffs, BMI Percentile	54.24	74.62	16.08	94.79	32	167	491	27	717	72.94%	28.86
		CR Cutoffs, Arm/Height	52.54	76.44	16.67	94.73	31	155	503	28	717	74.48%	28.99
Hispanic Females	Overall	BMI ≥ 85th Percentile	49.43	77.78	81.82	43.19	306	68	238	313	925	58.81%	27.21
		CR Cutoffs, BMI Percentile	31.50	92.16	89.04	39.94	195	24	282	424	925	51.57%	23.66
		CR Cutoffs, Arm/Height	33.28	88.24	85.12	39.53	206	36	270	413	925	51.46%	21.51
	TC	BMI ≥ 85th Percentile	46.45	63.60	38.77	70.54	321	507	886	370	2084	57.92%	10.06
		CR Cutoffs, BMI Percentile	29.52	79.90	42.15	69.56	204	280	1113	487	2084	63.20%	9.42
		CR Cutoffs, Arm/Height	27.06	80.55	40.83	69.00	187	271	1122	504	2084	62.81%	7.61
	LDL-C	BMI ≥ 85th Percentile	50.00	62.04	20.79	86.16	79	301	492	79	951	60.04%	12.04
		CR Cutoffs, BMI Percentile	34.18	79.32	24.77	85.81	54	164	629	104	951	71.82%	13.50
		CR Cutoffs, Arm/Height	29.75	80.71	23.50	85.22	47	153	640	111	951	72.24%	10.45
	HDL-C	BMI ≥ 85th Percentile	59.91	69.23	46.38	79.54	384	444	999	257	2084	66.36%	29.14
		CR Cutoffs, BMI Percentile	41.65	84.96	55.17	76.63	267	217	1226	374	2084	71.64%	26.62
		CR Cutoffs, Arm/Height	39.16	85.65	54.80	76.01	251	207	1236	390	2084	71.35%	24.81
	TG	BMI ≥ 85th Percentile	56.30	70.18	54.12	71.99	210	178	419	163	970	64.85%	26.48
		CR Cutoffs, BMI Percentile	37.27	85.93	62.33	68.67	139	84	513	234	970	67.22%	23.20
		CR Cutoffs, Arm/Height	33.24	86.60	60.78	67.49	124	80	517	249	970	66.08%	19.84
	SBP	BMI ≥ 85th Percentile	75.80	63.32	13.65	97.16	119	753	1300	38	2210	64.21%	39.12
		CR Cutoffs, BMI Percentile	53.50	79.01	16.31	95.69	84	431	1622	73	2210	77.19%	32.51
		CR Cutoffs, Arm/Height	55.41	80.47	17.83	95.93	87	401	1652	70	2210	78.69%	35.88
	DBP	BMI ≥ 85th Percentile	44.44	60.65	2.30	98.13	20	850	1310	25	2205	60.32%	5.09
		CR Cutoffs, BMI Percentile	33.33	76.90	2.92	98.23	15	499	1661	30	2205	76.01%	10.23
		CR Cutoffs, Arm/Height	31.11	78.15	2.88	98.20	14	472	1688	31	2205	77.19%	9.26
	HbA1C	BMI ≥ 85th Percentile	70.00	61.26	5.03	98.58	42	793	1254	18	2107	61.51%	31.26
		CR Cutoffs, BMI Percentile	51.67	77.63	6.34	98.21	31	458	1589	29	2107	76.89%	29.29
		CR Cutoffs, Arm/Height	55.00	79.04	7.14	98.36	33	429	1618	27	2107	78.36%	34.04
	FPG	BMI ≥ 85th Percentile	52.17	61.98	15.38	90.73	60	330	538	55	983	60.83%	14.16
		CR Cutoffs, BMI Percentile	33.91	78.69	17.41	89.99	39	185	683	76	983	73.45%	12.60
		CR Cutoffs, Arm/Height	31.30	80.53	17.56	89.85	36	169	699	79	983	74.77%	11.83

CR = cardiometabolic risk; Overall = unhealthy level on any CR variable; TC = total cholesterol; LDL-C = low-density lipoprotein cholesterol; HDL-C = high-density lipoprotein cholesterol; TG = triglycerides; SBP = systolic blood pressure; DBP = diastolic blood pressure; HbA1c = glycated hemoglobin; FPG = fasting plasma glucose. Unhealthy level of each CR variable includes both borderline-risk and high-risk levels, defined according to National Heart, Lung and Blood Institute Expert Panel Report 2012, American Academy of Pediatrics, and American Diabetes Association.

Sensi = sensitivity; Speci = specificity; PPV = positive predictive value; NPV = negative predictive value; Tr+ = true positives; Fal+ = false positives; Tr-= true negatives; Fal- = false negatives; Accur = Accuracy (i.e., % correctly classified); J = sensitivity +specificity –100 (i.e., Youden's Index)

Conventionally, BMI≥85thpercentile (overweight) was considered high-risk. For CR, BMI≥94thpercentile and arm/height≥0.19, were considered high-risk.

Appendix-2: Statistical Measures for Classification of Each Binary Cardiometabolic Risk Variable, Using Each Binary Measure of General Obesity, Reference Table for Males of Ages 12-18 by Race and Ethnicity, N=6,289

Race	CR	Predictor	Sensi	Speci	PPV	NPV	Tr+	Fal+	Tr-	Fal-	Total	Accur	J
Males of All Races/Ethnicities	TC	BMI ≥ 85th Percentile	48.79	67.57	39.53	75.23	850	1300	2709	892	5751	0.62	16.37
		CR Cutoffs, BMI Percentile	66.59	46.17	34.96	76.08	1160	2158	1851	582	5751	0.52	12.76
		CR Cutoffs, Arm/Height	60.96	54.38	36.73	76.22	1062	1829	2180	680	5751	0.56	15.34
	LDL-C	BMI ≥ 85th Percentile	54.22	68.45	29.52	85.99	289	690	1497	244	2720	0.66	22.67
		CR Cutoffs, BMI Percentile	69.79	46.87	24.25	86.42	372	1162	1025	161	2720	0.51	16.66
		CR Cutoffs, Arm/Height	66.42	54.14	26.09	86.87	354	1003	1184	179	2720	0.57	20.55
	HDL-C	BMI ≥ 85th Percentile	54.40	73.90	58.05	70.94	1248	902	2554	1046	5750	0.66	28.30
		CR Cutoffs, BMI Percentile	72.62	52.23	50.23	74.19	1666	1651	1805	628	5750	0.60	24.85
		CR Cutoffs, Arm/Height	66.48	60.47	52.75	73.10	1525	1366	2090	769	5750	0.63	26.95
	TG	BMI ≥ 85th Percentile	54.58	72.63	48.77	77.01	495	520	1380	412	2807	0.67	27.21
		CR Cutoffs, BMI Percentile	72.11	51.16	41.34	79.35	654	928	972	253	2807	0.58	23.26
		CR Cutoffs, Arm/Height	66.92	58.05	43.23	78.62	607	797	1103	300	2807	0.61	24.98
	SBP	BMI ≥ 85th Percentile	55.92	68.27	32.72	84.88	741	1524	3279	584	6128	0.66	24.19
		CR Cutoffs, BMI Percentile	74.79	47.51	28.22	87.23	991	2521	2282	334	6128	0.53	22.30
		CR Cutoffs, Arm/Height	68.68	55.57	29.89	86.54	910	2134	2669	415	6128	0.58	24.25
	DBP	BMI ≥ 85th Percentile	36.32	63.16	3.62	96.30	81	2158	3699	142	6080	0.62	-0.52
		CR Cutoffs, BMI Percentile	55.16	42.77	3.54	96.16	123	3352	2505	100	6080	0.43	-2.07
		CR Cutoffs, Arm/Height	52.47	50.54	3.88	96.54	117	2897	2960	106	6080	0.51	3.00
	HbA1C	BMI ≥ 85th Percentile	44.76	63.19	8.06	94.07	175	1996	3427	216	5814	0.62	7.95
		CR Cutoffs, BMI Percentile	63.68	42.80	7.43	94.23	249	3102	2321	142	5814	0.44	6.48
		CR Cutoffs, Arm/Height	56.01	50.23	7.51	94.06	219	2699	2724	172	5814	0.51	6.24
	FPG	BMI ≥ 85th Percentile	44.80	66.24	27.29	80.93	280	746	1464	345	2835	0.62	11.04
		CR Cutoffs, BMI Percentile	65.60	46.38	25.71	82.66	410	1185	1025	215	2835	0.51	11.98
		CR Cutoffs, Arm/Height	56.96	52.08	25.16	81.06	356	1059	1151	269	2835	0.53	9.04
Non-Hispanic White Males	Overall	BMI ≥ 85th Percentile	38.32	84.97	88.74	30.82	205	26	147	330	708	0.50	23.29
		CR Cutoffs, BMI Percentile	66.54	54.91	82.03	34.67	356	78	95	179	708	0.64	21.46
		CR Cutoffs, Arm/Height	65.05	60.12	83.45	35.74	348	69	104	187	708	0.64	25.16
	TC	BMI ≥ 85th Percentile	47.65	71.56	39.11	78.09	203	316	795	223	1537	0.65	19.21
		CR Cutoffs, BMI Percentile	67.37	50.05	34.09	80.00	287	555	556	139	1537	0.55	17.42
		CR Cutoffs, Arm/Height	61.97	58.15	36.21	79.95	264	465	646	162	1537	0.59	20.12
	LDL-C	BMI ≥ 85th Percentile	49.64	71.94	29.49	85.80	69	165	423	70	727	0.68	21.58
		CR Cutoffs, BMI Percentile	67.63	51.70	24.87	87.11	94	284	304	45	727	0.55	19.33
		CR Cutoffs, Arm/Height	66.19	56.63	26.51	87.63	92	255	333	47	727	0.58	22.82
	HDL-C	BMI ≥ 85th Percentile	48.17	78.67	66.09	63.75	343	176	649	369	1537	0.65	26.84
		CR Cutoffs, BMI Percentile	66.99	55.76	56.65	66.19	477	365	460	235	1537	0.61	22.75
		CR Cutoffs, Arm/Height	60.81	64.12	59.40	65.47	433	296	529	279	1537	0.63	24.94
	TG	BMI ≥ 85th Percentile	45.79	76.68	56.67	67.99	136	104	342	161	743	0.64	22.47
		CR Cutoffs, BMI Percentile	67.34	58.07	51.68	72.75	200	187	259	97	743	0.62	25.41
		CR Cutoffs, Arm/Height	61.62	61.43	51.55	70.62	183	172	274	114	743	0.62	23.05
	SBP	BMI ≥ 85th Percentile	53.80	71.45	30.80	86.75	170	382	956	146	1654	0.68	25.25
		CR Cutoffs, BMI Percentile	70.57	49.63	24.86	87.71	223	674	664	93	1654	0.54	20.20
		CR Cutoffs, Arm/Height	67.09	58.30	27.53	88.24	212	558	780	104	1654	0.60	25.38
	DBP	BMI ≥ 85th Percentile	46.03	67.24	5.30	96.90	29	518	1063	34	1644	0.66	13.27
		CR Cutoffs, BMI Percentile	58.73	46.11	4.16	96.56	37	852	729	26	1644	0.47	4.84
		CR Cutoffs, Arm/Height	65.08	54.21	5.36	97.50	41	724	857	22	1644	0.55	19.29
	HbA1C	BMI ≥ 85th Percentile	47.37	66.58	3.43	98.06	18	507	1010	20	1555	0.66	13.95
		CR Cutoffs, BMI Percentile	63.16	45.42	2.82	98.01	24	828	689	14	1555	0.46	8.58
		CR Cutoffs, Arm/Height	55.26	52.87	2.85	97.92	21	715	802	17	1555	0.53	8.13
	FPG	BMI ≥ 85th Percentile	36.75	69.01	25.21	79.33	61	181	403	105	750	0.62	5.75
		CR Cutoffs, BMI Percentile	59.64	50.34	25.45	81.44	99	290	294	67	750	0.52	9.98
		CR Cutoffs, Arm/Height	50.60	53.42	23.60	79.19	84	272	312	82	750	0.53	4.03

Continued on the Next Page.

Race	CR	Predictor	Sensi	Speci	PPV	NPV	Tr+	Fal+	Tr-	Fal-	Total	Accur	J
Non-Hispanic Black Males	Overall	BMI ≥ 85th Percentile	39.31	78.85	82.61	33.71	228	48	179	352	807	0.50	18.16
		CR Cutoffs, BMI Percentile	68.45	44.05	75.76	35.34	397	127	100	183	807	0.62	12.50
		CR Cutoffs, Arm/Height	69.83	50.22	78.19	39.45	405	113	114	175	807	0.64	20.05
	TC	BMI ≥ 85th Percentile	43.15	68.78	41.29	70.40	249	354	780	328	1711	0.60	11.94
		CR Cutoffs, BMI Percentile	60.49	45.41	36.05	69.31	349	619	515	228	1711	0.50	5.90
		CR Cutoffs, Arm/Height	53.38	55.20	37.75	69.94	308	508	626	269	1711	0.55	8.58
	LDL-C	BMI ≥ 85th Percentile	51.37	69.74	31.97	83.82	94	200	461	89	844	0.66	21.11
		CR Cutoffs, BMI Percentile	64.48	45.39	24.63	82.19	118	361	300	65	844	0.50	9.87
		CR Cutoffs, Arm/Height	60.11	54.77	26.89	83.22	110	299	362	73	844	0.56	14.87
	HDL-C	BMI ≥ 85th Percentile	56.90	73.13	45.11	81.39	272	331	901	206	1710	0.69	30.04
		CR Cutoffs, BMI Percentile	73.01	49.84	36.09	82.64	349	618	614	129	1710	0.56	22.85
		CR Cutoffs, Arm/Height	66.74	59.66	39.09	82.21	319	497	735	159	1710	0.62	26.40
	TG	BMI ≥ 85th Percentile	53.37	69.18	28.25	86.71	87	221	496	76	880	0.66	22.55
		CR Cutoffs, BMI Percentile	68.10	46.16	22.33	86.42	111	386	331	52	880	0.50	14.26
		CR Cutoffs, Arm/Height	65.64	55.79	25.24	87.72	107	317	400	56	880	0.58	21.43
	SBP	BMI ≥ 85th Percentile	51.42	71.90	37.88	81.62	236	387	990	223	1836	0.67	23.31
		CR Cutoffs, BMI Percentile	73.64	50.76	33.27	85.24	338	678	699	121	1836	0.56	24.40
		CR Cutoffs, Arm/Height	64.49	59.91	34.91	83.50	296	552	825	163	1836	0.61	24.40
	DBP	BMI ≥ 85th Percentile	27.27	65.92	3.41	95.35	21	594	1149	56	1820	0.64	-6.81
		CR Cutoffs, BMI Percentile	48.05	44.52	3.69	95.10	37	967	776	40	1820	0.45	-7.43
		CR Cutoffs, Arm/Height	44.16	53.93	4.06	95.63	34	803	940	43	1820	0.54	-1.91
	HbA1C	BMI ≥ 85th Percentile	43.17	66.25	16.17	88.54	98	508	997	129	1732	0.63	9.42
		CR Cutoffs, BMI Percentile	61.23	44.52	14.27	88.39	139	835	670	88	1732	0.47	5.75
		CR Cutoffs, Arm/Height	51.54	53.22	14.25	87.93	117	704	801	110	1732	0.53	4.76
	FPG	BMI ≥ 85th Percentile	45.97	67.28	18.51	88.51	57	251	516	67	891	0.64	13.24
		CR Cutoffs, BMI Percentile	67.74	45.89	16.83	89.80	84	415	352	40	891	0.49	13.64
		CR Cutoffs, Arm/Height	59.68	54.24	17.41	89.27	74	351	416	50	891	0.55	13.91
Hispanic Males	Overall	BMI ≥ 85th Percentile	46.80	80.49	89.58	29.68	344	40	165	391	940	0.54	27.29
		CR Cutoffs, BMI Percentile	73.88	48.29	83.67	34.02	543	106	99	192	940	0.68	22.17
		CR Cutoffs, Arm/Height	73.20	51.22	84.33	34.77	538	100	105	197	940	0.68	24.42
	TC	BMI ≥ 85th Percentile	54.60	62.95	38.26	76.73	344	555	943	286	2128	0.60	17.55
		CR Cutoffs, BMI Percentile	72.38	42.32	34.55	78.47	456	864	634	174	2128	0.51	14.70
		CR Cutoffs, Arm/Height	67.30	49.60	35.96	78.29	424	755	743	206	2128	0.55	16.90
	LDL-C	BMI ≥ 85th Percentile	61.14	63.80	27.23	88.11	107	286	504	68	965	0.63	24.94
		CR Cutoffs, BMI Percentile	77.71	42.15	22.93	89.52	136	457	333	39	965	0.49	19.87
		CR Cutoffs, Arm/Height	74.29	49.75	24.67	89.73	130	397	393	45	965	0.54	24.03
	HDL-C	BMI ≥ 85th Percentile	57.65	70.91	62.85	66.23	565	334	814	415	2128	0.65	28.56
		CR Cutoffs, BMI Percentile	76.94	50.70	57.12	72.03	754	566	582	226	2128	0.63	27.64
		CR Cutoffs, Arm/Height	70.71	57.67	58.78	69.76	693	486	662	287	2128	0.64	28.38
	TG	BMI ≥ 85th Percentile	61.71	73.12	60.34	74.24	245	161	438	152	996	0.69	34.83
		CR Cutoffs, BMI Percentile	78.09	49.75	50.74	77.40	310	301	298	87	996	0.61	27.84
		CR Cutoffs, Arm/Height	72.04	56.26	52.19	75.22	286	262	337	111	996	0.63	28.30
	SBP	BMI ≥ 85th Percentile	60.50	62.42	30.35	85.38	288	661	1098	188	2235	0.62	22.93
		CR Cutoffs, BMI Percentile	78.15	41.96	26.70	87.65	372	1021	738	104	2235	0.50	20.11
		CR Cutoffs, Arm/Height	73.95	49.29	28.30	87.49	352	892	867	124	2235	0.55	23.24
	DBP	BMI ≥ 85th Percentile	37.88	57.64	2.67	96.80	25	912	1241	41	2219	0.57	-4.48
		CR Cutoffs, BMI Percentile	65.15	37.95	3.12	97.26	43	1336	817	23	2219	0.39	3.10
		CR Cutoffs, Arm/Height	54.55	44.50	2.92	96.96	36	1195	958	30	2219	0.45	-0.96
	HbA1C	BMI ≥ 85th Percentile	48.91	57.98	4.95	96.21	45	864	1192	47	2148	0.58	6.89
		CR Cutoffs, BMI Percentile	68.48	38.23	4.73	96.44	63	1270	786	29	2148	0.40	6.71
		CR Cutoffs, Arm/Height	66.30	45.04	5.12	96.76	61	1130	926	31	2148	0.46	11.34
	FPG	BMI ≥ 85th Percentile	49.13	62.01	34.14	75.25	141	272	444	146	1003	0.58	11.14
		CR Cutoffs, BMI Percentile	69.34	41.48	32.20	77.14	199	419	297	88	1003	0.49	10.82
		CR Cutoffs, Arm/Height	60.98	46.93	31.53	75.00	175	380	336	112	1003	0.51	7.90

CR = cardiometabolic risk; Overall = unhealthy level on any CR variable; TC = total cholesterol; LDL-C = low-density lipoprotein cholesterol; HDL-C = high-density lipoprotein cholesterol; TG = triglycerides; SBP = systolic blood pressure; DBP = diastolic blood pressure; HbA1c = glycated hemoglobin; FPG = fasting plasma glucose. Unhealthy level of each CR variable includes both borderline-risk and high-risk levels, defined according to National Heart, Lung and Blood Institute Expert Panel Report 2012, American Academy of Pediatrics, and American Diabetes Association.

Sensi = sensitivity; Speci = specificity; PPV = positive predictive value; NPV = negative predictive value; Tr+ = true positives; Fal+ = false positives; Tr-= true negatives; Fal- = false negatives; Accur = Accuracy (i.e., % correctly classified); J = sensitivity +specificity –100 (i.e., Youden's Index)

Conventionally, BMI≥85^{th}percentile (overweight) was considered high-risk. For CR, BMI≥64^{th}percentile and arm/height≥0.16, were considered high-risk.
